# Review of potential risks associated with supplemental dietary exposure to nitrate-containing compounds in swine—a paradox in light of emerging benefits

**DOI:** 10.1093/tas/txab203

**Published:** 2021-10-18

**Authors:** Candace L Doepker, Melissa M Heintz, Jennifer van de Ligt, Daniele S Wikoff

**Affiliations:** 1 ToxStrategies, Inc., Newport, KY 41071, USA; 2 ToxStrategies, Inc., Asheville, NC 28801, USA; 3 ToxStrategies, Inc., Brooklyn Park, MN 55443, USA; 4 Department of Veterinary Population Medicine, College of Veterinary Medicine, University of Minnesota, St. Paul, MN 55108, USA

**Keywords:** methemoglobin, methemoglobinemia, nitrate, review, safety, swine

## Abstract

Calcium nitrate has been reported to benefit reproductive outcomes in sows and their offspring when administered via the feed (15 to 19 mg/kg-body weight [bw]/day) during the periparturient period. Traditionally, dietary nitrate had been considered a methemoglobinemia (MetHb) risk in swine. Similar hazard concerns have existed in humans, but a recent benefit/risk analysis established that nitrate levels associated with well-recognized health benefits outweigh potential risks. A similar benefit/risk perspective in swine was lacking and challenged by sparse published hazard data, often referenced within larger reviews related to all livestock. The objective of this review was to better characterize the potential for adverse health and performance effects reported in the literature for swine consuming nitrate and to provide metrics for evaluating the reliability of the studies reviewed. Supplemental exposure via feed or drinking water was considered for any life stage, dose, and exposure duration. More than 30 relevant studies, including case reports and reviews, examined calcium, potassium, sodium, or unspecified nitrate salts at doses up to 1,800 mg nitrate/kg-bw/day for exposures ranging from 1 to 105 d. The studies primarily evaluated weight gain, blood methemoglobin levels, or vitamin A homeostasis in sows or growing swine. An extensive review of the literature showed reports of adverse effects at low nitrate doses to be of low reliability. Conversely, reliable studies corroborate nitrate intake from feed or drinking water at levels equal to or greater than the European Food Safety Authority’s no-observed-adverse-effect level (NOAEL) for swine of 410 mg nitrate/kg-bw/day, with no MetHb or other adverse effects on reproduction, growth, or vitamin A levels. Using a weight-of-evidence evaluation, we have moderate-to-high confidence that the NOAEL for nitrate supplementation in swine is likely between 600 and 800 mg/kg-bw/day. These levels are several-fold higher than dietary nitrate concentrations (19 mg/kg-bw/day) that are known to benefit birth outcomes in sows. This review elucidates the quality and reliability of the information sources historically used to characterize nitrate in swine feed as a contaminant of concern. Results from this evaluation can assist risk managers (e.g., regulatory officials and veterinarians) in consideration of proposed benefits as well as reassuring swine producers that low-level nitrate supplementation is not anticipated to be a concern.

## INTRODUCTION

A rapidly growing body of scientific evidence has evaluated nitrate in the context of human health benefits (Bryan and Loscalzo, 2017). The production of nitric oxide following the oral consumption of nitrate is believed to be the mechanism for the most well-established benefit to human health of improved cardiovascular health (reviewed by Wikoff et al., 2018). Nitric oxide is typically formed by oral bacteria, which reduce nitrate to nitrite and nitrite to nitric oxide. Nitrate in farm animals (i.e., livestock) has also been used to reduce methane emissions in cattle (e.g., Lee and Beauchemin, 2014) and as a diuretic in pigs, cattle, and horses (EMEA, 1997). Recently, calcium nitrate has been evaluated as a beneficial nitrate source in animal feed to improve reproductive outcomes in sows and their offspring when administered on gestation day (GD) 108 prior to farrowing and through lactation day (LD) 5 (van den Bosch et al., 2019a, 2019b). Most piglet mortality during farrowing is due to broken umbilical cords or prolonged parturition duration, and results of this study indicate that maternal low-dose nitrate supplementation lowers the risk of piglet loss during farrowing (van den Bosch et al., 2019a, 2019b).

The main risk from repeated exposure to high concentrations of nitrate is methemoglobinemia (MetHb; Mensinga et al., 2003). Methemoglobin is produced after nitrate is converted to nitrite. Nitrite reacts with ferrous hemoglobin, the primary transporter of oxygen in the blood, to form ferric hemoglobin (methemoglobin and nitric oxide). Ferric hemoglobin does not bind oxygen well, thus altering oxygen transport. MetHb manifests when tissues are significantly deprived of oxygen (Mensinga et al., 2003; EFSA, 2020). In an evaluation of nitrate toxicity in humans, Wikoff et al. (2018) reported that concerns regarding MetHb have been recently recognized to be confounded by high levels of bacterial contamination in drinking water (USEPA, 1991; ATSDR, 2017; OEHHA, 2018; Wikoff et al., 2018). For example, nitrate levels in drinking water were thought to cause MetHb in infants; however, exposure to high bacterial loads in drinking water was later determined to be a more likely cause of MetHb (Wikoff et al., 2018).

The safety of nitrate exposure has also been questioned due to indications of MetHb in swine, and these concerns have led to the common belief that nitrate is detrimental to swine health (Smith et al., 1959; Emerick, 1974; Bruning-Fann and Kaneene, 1993; Nyachoti and Kiarie, 2010). This risk of MetHb is often mentioned but not clearly discussed in studies examining the risks or benefits of nitrate supplementation in animals. The understanding of MetHb risk in swine is complicated further by inconsistent study reporting factors, making dose comparisons across studies difficult. These inconsistencies include the administration of different nitrate salts (lack of data normalization), differences in study design with respect to oral route of exposure (i.e., feed or drinking water), age at onset, duration of exposure, and confounding factors such as microbiological load in drinking water. As a result, currently published literature reviews on nitrate in swine are challenging to decipher with regard to what concentrations of nitrate may pose adverse-effect concerns in swine. In a preliminary review of the literature regarding nitrate supplementation and swine health, four reviews were identified. These relevant reviews are generally older, include data for other livestock species, emphasize old case reports and anecdotal information in swine, or include abbreviated research summaries (Smith et al., 1959; Emerick, 1974; Bruning-Fann and Kaneene, 1993; Nyachoti and Kiarie, 2010). Examples of reported effects in swine from these previous reviews include:

- Labored breathing, pulmonary edema, convulsion, mortality, and late-term abortions after ingestion of wet oat and wheat straw-induced acute poisoning (as reviewed by Smith et al., 1959). The specific nitrate doses were not reported.
- Decreased feed consumption and weight gain, no effects on reproduction, and reduced utilization of β-carotene at very high feed concentrations, leading the author to recommend a safe level of 0.1% nitrate-nitrogen in feed across livestock species, including swine. The authors noted nitrite and MetHb concerns (as reviewed by Emerick, 1974).
- Decreased feed consumption (at greater than 1.84% nitrate in feed), severe gastritis (large, acute nitrate doses, exposure route not specified), impaired thyroid (doses and exposure route not specified), interference with vitamin A metabolism (0.04% and 0.3% nitrate, exposure route not specified), and hematologic changes, including MetHb (doses and exposure route not specified) (as reviewed by Bruning-Fann and Kaneene, 1993).
- Increased respiration rate, diarrhea, reduced feed intake, poor growth, increased abortion frequency among sows, and reduced vitamin A utilization have been reported in swine exposed to nitrate in drinking water. The specific nitrate doses attributed to these adverse effects were not reported; however, the authors presented a recommended nitrate-nitrogen limit of 100 ppm in drinking water (as reviewed by Nyachoti and Kiarie, 2010).

Based on the reported effects described above, it was difficult to draw concise conclusions on no-observable-adverse-effect levels (NOAELs) from these reviews and subsequent interpretations of safety.

Nitrite presence in livestock feed as a possible hazard was reviewed by the European Food Safety Authority (EFSA) in 2009, wherein the formation of MetHb was recognized as a concern. However, in a subsequent review, nitrate was deemed to have a low order of toxicity compared with nitrite in swine (EFSA, 2020). EFSA identifies a NOAEL of 410 mg nitrate per kilogram body weight per day (mg/kg-body weight [bw]/day) for pigs in their review. This NOAEL is based on a review of only a few published reports (Seerley et al., 1965; Wood et al., 1967; van den Bosch et al., 2019a, 2019b), with heavy reliance on the work of Wood et al. (1967) to determine the point of departure (POD). EFSA concluded that although a limited number of studies were available for review, the risk of adverse health effects from feeds containing nitrate is very low. This authoritative position is helpful, but if one were considering increasing sow nitrate supplementation for piglet livability benefits, the use of currently published reviews and anecdotal reports as references may raise questions of safety, especially in the midst of long-standing beliefs around nitrate toxicity.

To assess the safety of nitrate exposure in swine, a stepwise, weight-of-evidence (WOE) evaluation was used to identify relevant studies and reliable NOAELs for nitrate administration. Results from the comprehensive review presented herein provide a complete risk profile of nitrate consumption in swine as well as a robust comparison of appropriate NOAELs to proposed beneficial supplementation levels. In addition, conclusions from this review further support EFSA’s NOAEL of 410 mg/kg-bw/day.

## MATERIALS AND METHODS

### Identification of Relevant Studies

PubMed, Embase, and the public domain were searched for any study evaluating oral nitrate exposure in swine. The date of the last literature search was conducted on October 13, 2020. Literature search terms and example search syntax (specific to Embase):

‘swine nitrate feed’ OR ((‘swine’/exp OR swine) AND (‘nitrate’/exp OR nitrate) AND feed)’;‘swine nitrate drinking w’ OR ((‘swine’/exp OR swine) AND (‘nitrate’/exp OR nitrate) AND (‘drinking’/exp OR drinking) AND ‘water’/exp OR water))’;’pig nitrate drinking water’ OR ((‘pig’/exp OR pig) AND (‘nitrate’/exp OR nitrate) AND (‘drinking’/exp OR drinking) AND (‘water’/exp OR water))’’pig nitrate feed’ OR ((‘pig’/exp OR pig) AND (‘nitrate’/exp OR nitrate) AND feed)’

All studies investigating swine and supplemental nitrate exposure via feed or drinking water were considered relevant, along with any nitrate salt forms, life stage, dose, and exposure duration. Case reports and review articles encompassing any relevant criteria were also included. Studies were excluded if they did not include swine exposures, did not test nitrate, did not examine oral exposure, or if the study only tested nitrite without also testing nitrate.

Hand-searching and gray literature searching (beyond the searching restricted to the syntax above) were also conducted. This searching was targeted to include authoritative databases, such as the European Food Safety Authority (EFSA), Joint World Health Organization/Food and Agricultural Organization Expert Committee on Food Additives (JECFA), Scientific Committee on Food (SCF), International Agency for Research on Cancer (IARC), U.S. Environmental Protection Agency (USEPA), and U.S. Food and Drug Administration (FDA) Center for Veterinary Medicine (CVM). In addition, primary articles identified via hand-searching were cross-checked against literature search results.

The literature search was not restricted to English-language studies. Non-English studies were translated if an initial determination suggested that safety or toxicology parameters may have been investigated. Similarly, co-exposure studies, in which a nitrate compound was administered with another agent, were also reviewed to assess their relevance to the overall objective. Our approach with reviews was to use them as a baseline understanding of potential risks (Smith et al., 1959; Emerick, 1974; Bruning-Fann and Kaneene, 1993; Nyachoti and Kiarie, 2010).

### Assessment of Study Quality

After studies were identified for inclusion as described above, the quality of each study was documented. Each primary study was examined for reliability and quality using established and well-recognized criteria and metrics described by Klimisch et al. (1997). The standard Klimisch criteria were modified slightly, as no standard testing guidelines (e.g., Organisation for Economic Co-operation and Development [OECD]) or requirements (e.g., Good Laboratory Practices [GLP]) for study design or data reporting exist for safety studies in swine or livestock in general. Thus, rather than requiring studies to meet an internationally accepted testing guideline, or preferably, to be performed according to GLP, study quality was designated according to the criteria presented in Table 1. Review articles were excluded from the Klimisch scoring step.

**Table 1.** Modified Klimisch criteria used in the present review compared with original criteria

Score	Original criteria according to Klimisch et al. (1997)	Modified for present review
1. Reliable without Restriction	Studies or data carried out or generated according to generally valid and/or internationally accepted testing guidelines (preferably performed according to GLP) or in which the test parameters documented are based on a specific (national) testing guideline (preferably performed according to GLP) or in which all parameters described are closely related/comparable to a guideline method.	Studies carried out according to generally valid principles; data reporting/documentation is sufficient for an assessment and expert judgment
2. Reliable with Restrictions	Studies or data from the literature, reports of studies (mostly not performed according to GLP), in which the test parameters documented do not totally comply with the specific testing guideline but are sufficient to accept the data or in which investigations are described that cannot be subsumed under a testing guideline but which are nevertheless well documented and scientifically acceptable.	Studies carried out according to generally valid principles; data reporting/ documentation may be limited and thus may affect expert judgment
3. Not reliable	Studies or data from the literature/reports in which there are interferences between the measuring system and the test substance, or in which organisms/test systems were used that are not relevant in relation to the exposure (e.g., unphysiological pathways of application) or that were carried out or generated according to an unacceptable method, the documentation of which is not sufficient for an assessment and that is not convincing for an expert judgment.	Data reporting/documentation is not sufficient for an assessment or for an expert judgment (e.g., lack of reporting on statistical significance and/or dose levels affected, or co-exposure with known toxicant without a group exposed to nitrate alone)
4. Not assignable	Studies or data from the literature that do not give sufficient experimental details and that are listed only in short abstracts or secondary literature (books, reviews, etc.).	Abstract-only studies—published abstracts for which a full publication was not identified

### Dose Conversions

To compare lowest-observed-adverse-effect-levels (LOAELs) and NOAELs among studies, it was necessary that nitrate exposures be expressed in a similar metric. In toxicology, LOAELs and NOAELs are based on intake and expressed as mg/kg-bw/day. Many of the studies did not report findings using this metric; therefore, various conversion calculations were required so that all administered doses of nitrate in feed or drinking water could be compared (mg/kg-bw/day). Additionally, studies using different salt forms of nitrate were converted to “as nitrate” using appropriate physical and chemical properties (Table 2). Most of the swine feeding studies were conducted in gestating and lactating sows or growing swine. The tested concentrations were typically reported as a percent of a final formulation or in parts per million (ppm) of the nitrate salt in feed. The tested concentrations were converted to mg/kg-bw/day nitrate by first adjusting for molecular weight (MW), based on the salt form of nitrate administered, and then applying a dosimetric adjustment factor based on National Research Council (NRC, 2012) parameters for BW and feed intake. When the authors did not report exact values, we assumed that a percentage of body weight is consumed in feed per day using the NRC values. For drinking water studies, the tested concentrations were similarly converted to mg nitrate/kg-bw/day, as described for feed—first by adjusting for MW, based on the salt form of nitrate administered, and then applying a dosimetric adjustment factor assuming a volume of drinking water intake per day (L/kg-bw/day). Supporting calculations for all studies are provided in Supplementary Information.

**Table 2.** Physical and chemical properties for nitrate salts investigated in swine (Kim et al., 2019)

	Calcium nitrate	Potassium nitrate	Sodium nitrate	Nitrate ion
CAS#	10124-37-5	7757-79-1	7631-99-4	14797-55-8
Structure				
Molecular formula	Ca(NO_3_)_2_	KNO_3_	NaNO_3_	NO_3_−
MW	164.09 g/mol	101.103 g/mol	84.995 g/mol	62.005 g/mol
Water solubility	121.2 g/100 mL	35 g/100 mL	730 mg/mL at 0 °C	Not reported
MW adjustment factor^*a*^	0.38	0.61	0.73	Not applicable

MW = molecular weight.

^*a*^Administered concentration of nitrate salt was multiplied by the MW adjustment factor (MW nitrate salt/MW nitrate) to obtain the administered concentration of nitrate.

### WOE Evaluation

EFSA has suggested previously that swine may safely consume up to 410 mg/kg-bw/day nitrate through feed or vegetation sources. However, EFSA selected this value based on a small number of studies. The goal of this work was to use a WOE approach to determine: 1) whether this EFSA value is reliable and 2) if the nitrate levels administered to periparturient sows (19 mg/kg-bw/d; van den Bosch et al., 2019a, 2019b) for benefit in terms of piglet livability are at or near levels associated with adverse health effects (in particular MetHb), as reported in the literature.

The first step in applying the WOE approach was to look at gathered information individually. We reviewed each included study and documented, based on the authors’ conclusions (to limit reviewer bias), the study objectives, and the endpoints reported in the paper’s Results section. Exposure concentrations were documented based on authors’ designations or appropriate normalization conversion metrics (Table 2). For each study, exposure duration and adverse effects were determined, if possible. The lowest dose level associated with the adverse effects was identified as the LOAEL. Exposure levels where no adverse effects were observed were designated as NOAELs. Examples of adverse effects include changes in performance (e.g., weight loss, decreased food intake) or overall health effects (e.g., mortality, vitamin A levels). Health effects of particular interest included nitrate blood levels, methemoglobin levels, and any occurrence of MetHb.

As previously described, the application of the modified Klimisch criteria was used for transparent and consistent documentation of study quality and reliability. Consequently, studies with Klimisch scores of 1 and 2 were given more weight in the overall evaluation and used to compare with nitrate levels in feed established by van den Bosch et al. (2019a, 2019b; 19 mg/kg-bw/day) and EFSA (2020; 410 mg/kg-bw/day). In addition, the variance around reliable NOAELs was also considered.

## RESULTS

### Identification and Inclusion of Relevant Literature for WOE Evaluation

A total of 29 studies were identified, including 4 review articles and 2 authoritative reviews (Smith et al., 1959; Emerick, 1974; Bruning-Fann and Kaneene, 1993; EFSA, 2009, 2020; Nyachoti and Kiarie, 2010). The four review articles were used to establish background and context for purported adverse events, and the EFSA (2020) review was used to evaluate the established health-based benchmark of 410 mg/kg-bw/day. Using the inclusion and exclusion criteria as described above, 21 of the 29 studies qualified for full-text review (Supplementary Table S1). 

Following full-text review, three studies were excluded from the WOE assessment, bringing the total to 18 studies. Gwatkin and Plummer (1946) conducted a gavage study aimed at understanding the acute toxicity associated with the delivery of one bolus dose of potassium nitrate. The authors noted significant damage from the gavage procedure itself, therefore limiting the ability to distinguish treatment-related adverse effects. Bjornsen et al. (1961) reviewed case reports of potassium nitrate contamination in swine drinking water in wells. However, the authors were not able to identify which wells the affected animals drank from; as such, they could not determine the concentrations of potassium nitrate exposure to the swine. Persson et al. (1988) examined potential mutagenic effects of high nitrate levels in swine drinking water in bacteria treated with swine urine, lymphocytes, and bone marrow cells. Although no evidence of mutagenicity was observed at any of the doses tested, extrapolation of in vitro to in vivo effects was difficult to interpret. The lack of definitive conclusions prevented the derivation of LOAEL or NOAEL values for these studies. As a result, studies by Gwatkin and Plummer (1946), Bjornsen et al. (1961), and Persson et al. (1988) were excluded from further evaluation.

In addition to the articles identified in the literature search, two non-published studies provided by a feed ingredient supplier (Cargill, Inc., 2017, 2020; submitted as a companion article to this publication in *Translational Animal Science*, see van de Ligt et al. (2021) were also considered as part of the WOE exercise, bringing the total number of studies included in the WOE evaluation to 20. Each study was assessed for reliability using the modified Klimisch scoring (Table 1). 

### Overview of the Relevant Literature

Out of the 20 studies included in the WOE evaluation, 8 investigated nitrate effects via administration in drinking water and the other 12 studies examined nitrate supplementation in feed (Supplementary Table S1). The extent of data reporting and level of detail varied widely, from peer-reviewed journal articles investigating intentional supplementation with nitrate to case reports with abbreviated reporting of information. None of the studies were conducted under GLP designation; therefore, the modified Klimisch scoring was valuable for evaluating confidence in the data reported. The highest confidence score (Klimisch 1) was given to four published reports (Bruning-Fann et al., 1996; Trevisi et al., 2011; van den Bosch et al., 2019a, 2019b) and the two unpublished reports (Cargill, Inc., 2017, 2020), whereas 9 of the published studies received a modified Klimisch designation of “2” due to some limitations in data reporting. One study received a modified Klimisch score of 3 due to insufficient data reporting (Paulson and Aschbacher, 1990), and the four oldest studies received a modified Klimisch score of 4 since they were only published as abstracts (Case, 1957; Garner et al., 1958; Tollett et al., 1960; Koch et al., 1963). The studies with higher-quality designations (modified Klimisch 1 and 2) were weighted more heavily and guided the decision-making in the WOE evaluation.

The duration of exposure varied greatly across studies, with nitrate exposures ranging from 1 wk (e.g., in feed; Leĭtis and Emel′ianov, 1969) to 125 d (e.g., in drinking water; Seerley et al., 1965). Five of the studies reviewed as part of the WOE exercise examined nitrate exposures for unspecified periods of time, but more than half of the studies evaluated examined nitrate exposure for at least 3 wk. Seven studies investigated longer-term exposures for greater than 8 wk (Garner et al., 1958; Koch et al., 1963; Seerley et al., 1965; Garrison et al., 1966; Wood et al., 1967; Hutagalung et al., 1968; Bouwkamp and Counotte, 1988). In addition, several studies monitored for treatment-related effects throughout the study duration (e.g., Seerley et al., 1965; Leĭtis and Emel′ianov, 1969; Anderson and Stothers, 1978; Jahreis et al., 1987).

Out of the studies included in the WOE evaluation, nitrate was tested as the calcium, potassium, sodium, or an unspecified counterion in exposure scenarios commonly involving administration of nitrate to sows, during late gestation or early lactation, or to growing swine (Supplementary Table S1). Additionally, 8 of the 20 studies used in the WOE evaluation were co-exposure studies in which a nitrate compound was administered with another agent. One such study involved co-exposure with sulfate, with no nitrate-only comparison group (Anderson and Stothers, 1978). It is important to note that sulfate may also interact with hemoglobin to induce sulfhemoglobinemia (Michel, 1938). Additionally, two other studies examined the effects of nitrate co-administration with a sulfa antibiotic (Paulson and Aschbacher, 1990) or iodine (Jahreis et al., 1986). Five studies also examined supplemental exposure of nitrate with vitamin A or its precursor, β-carotene (Tollett et al., 1960; Koch et al., 1963; Garrison et al., 1966; Wood et al., 1967; Hutagalung et al., 1968).

The studies evaluated using the WOE approach examined a wide array of safety endpoints across nitrate exposure, with doses spanning nearly 900-fold (~2 to ~1,800 mg nitrate/kg-bw/day; Tollett et al., 1960; Bruning-Fann et al., 1996). This range reflects doses after adjusting for MW based on the form of nitrate administered as well as conversions to compare feed to drinking water concentrations (see Supplementary Information). The estimated nitrate in mg/kg-bw/day for drinking water studies does not include any background nitrate that may also have been present. Feed formulation may also contain background levels of nitrate, but this was not identified by any study authors as a defined (quantified) contributing source and, thus, could not be accounted for in our review.

### Potential Nitrate Risks Identified from the Literature Review

As described in the Introduction section, potential adverse effects have been associated with increased nitrate exposure in swine. Common endpoints evaluated by the authors in the 20 studies reviewed herein were grouped into four main categories. Methemoglobin was the most commonly investigated endpoint by authors, followed by vitamin A levels in the liver or serum, growth performance, and reproductive performance. The review of specific study endpoints and the comparison of results across studies enabled the distinction between clinical changes (e.g., increases in blood methemoglobin) vs. the manifestation of an adverse outcome, such as development of MetHb, as well as the corresponding nitrate exposure levels of these observed effects. When discussing methemoglobin changes, it is important to note that changes in blood methemoglobin may be discussed more properly as a clinical change, rather than an adverse effect, until toxicity or signs of adversity manifest into pathological deviations (Bhattarai et al., 2019). Methemoglobin analysis is a reliable indicator of excess nitrate or nitrite exposure by itself only under conditions of acute toxicity (Thompson, 2014). The Merck Index in swine reports a typical hemoglobin range of 6.21 to 9.93 mmol/L. If methemoglobin were to increase, this range would be expected to decrease. In humans, Wright et al. (1999) suggest that the acute toxicity resulting from the presence of methemoglobin has a threshold of greater than 10% methemoglobin in the blood, and this threshold is expected to be similar in swine. For the evaluation presented below, the authors’ designation of oxygen-related blood parameters was relied upon. Thus, if the authors did not indicate that hemoglobin ratios were altered, or that methemoglobin was increased in the blood, results were noted in support of an absence of methemoglobin adverse effects.

### Integrated WOE for Nitrate Exposure in Swine

**Figure 1.**
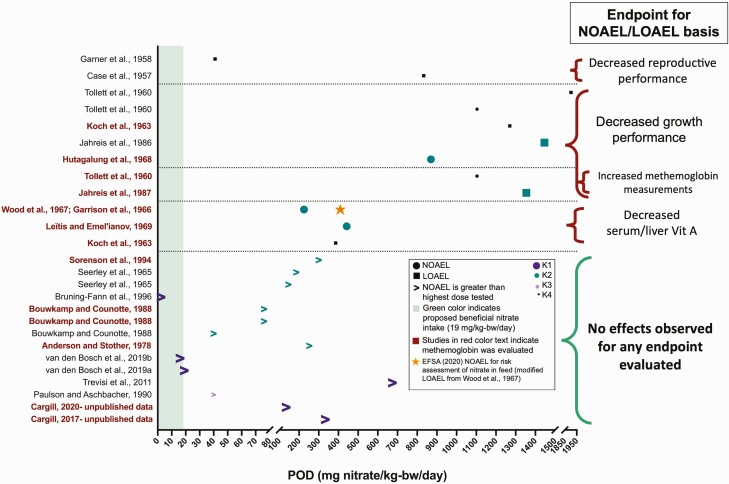
Comparison of PODs from studies investigating nitrate exposure in swine. Identified PODs from studies included in the WOE evaluation. If both a NOAEL and a LOAEL were determined for an individual study, the NOAEL was used. Modified Klimisch scores (K1 to K4) are indicated by the size of the data point in the figure.

The NOAELs (and LOAELs in the absence of a NOAEL) identified from the 20 studies in the literature review were plotted for comparison between studies in Fig. 1. In addition, modified Klimisch scores associated with each study were indicated for each POD to aid in overall judgment. For comparison purposes, the NOAEL previously determined by EFSA (2020) based on the study by Wood et al. (1967) is also included in Fig. 1 (410 mg/kg-bw/day). In instances where statistical significance was not indicated, NOAELs or LOAELs were identified based on the study authors’ reported effects, which may not be toxicologically significant but represent observed adverse physiological changes. If no adverse effects were observed for the endpoints evaluated, this valuable information was noted as a NOAEL with a greater than (>) sign in Fig 1, indicating no adverse effects observed, and, therefore, the effect level is higher than the highest dose investigated.

The majority of studies fell into modified Klimisch scores of 1 or 2 (K1 or K2), and because of the greater confidence in these studies, their results drove the overall conclusions. For each endpoint category characterized below, perspective was provided by the EFSA benchmark of 410 mg/kg-bw/day.

#### No effects observed for any endpoint, including no evidence of increased methemoglobin.

Twelve studies had no effects observed for any endpoint measured, and the majority of these studies were designated as K1 or K2 quality scores. Trevisi et al. (2011; K1) reported no adverse effects at 674 mg/kg-bw/day when animals were exposed to nitrate for 2 wk. This value is higher than the EFSA benchmark of 410 mg/kg-bw/day, although the treatment duration is shorter than the 84-d study (Wood et al., 1967) utilized by EFSA to derive their benchmark value. The authors did not measure methemoglobin because the focus of the work was on saliva levels of nitrate. It is reasonable, however, to assume that any difficulty breathing or MetHb-like symptoms would certainly have been noted by the authors.

Five of the 12 studies with no observed effects measured methemoglobin in the blood, with none of them reporting increased methemoglobin levels following nitrate exposure nor did they find any evidence of MetHb (Anderson and Stothers, 1978; Bouwkamp and Counotte, 1988; Sørensen et al., 1994; Cargill, Inc., 2017, 2020). The highest concentration of nitrate exposure in these five studies was the Cargill, Inc. (2017) study, at 334 mg/kg-bw/day, suggesting that the NOAEL is actually greater than this value. The next-highest exposure level in this group of five was the Sørensen et al. (1994) work, which looked at effects following a 6-wk exposure to potassium nitrate in drinking water. No evidence of MetHb or effects on growth, feed intake/utilization, or water consumption were observed after the 6 wk of exposure to ~300 mg/kg-bw/day nitrate beginning after weaning (Sørensen et al., 1994). This longer exposure duration adds confidence that the NOAEL is greater than 300 mg/kg-bw/day. Collectively, these data support the existing EFSA benchmark of 410 mg/kg-bw/day and even suggest a low likelihood of concern of nitrate levels up to 674 mg/kg-bw/day (as determined by Trevisi et al., 2011).

#### Increased methemoglobin.

Only two studies reported increases in methemoglobin (Tollet et al., 1960; Jahreis et al., 1987). Both exposures were on the order of 1 g/kg-bw/day, and both were co-exposure studies. Jahreis et al. (1987; co-administered iodine) found that a 6-wk exposure to nitrate in piglets led to an increase in methemoglobin, but the units of measurement were not reported. Despite statistical significance in an increase between controls and piglets receiving dietary nitrate supplementation for 6 weeks (LOAEL = 1.35 g/kg-bw/day), there was no mention of concern for outward manifestation of MetHb. Thus, the endpoint is noted as increased methemoglobin levels, not adversity. Tollett et al. (1960) had similar findings (LOAEL = 1.9 g/kg-bw/day), but this study was assigned a K4 value, thus given less weight, as it was published only as an abstract. However, the authors also reported an increase in methemoglobin without providing specifics as to level and offered no evidence of adversity (signs of MetHb). These results further suggest that higher nitrate levels than the EFSA benchmark value of 410 mg/kg-bw/day may be associated with increased methemoglobin levels in the blood but not with adversity manifested as MetHb concern.

#### Decreased serum or liver vitamin A.

The presence of nitrite in the blood can lead to depletion of vitamin A stores. This is thought to occur through an impact of nitrite on the thyroid, which, in turn, affects the ability of the thyroid to convert carotene to vitamin A. For this reason, a few studies evaluating exposure to nitrate also co-administered vitamin A or β-carotene (Tollett et al., 1960; Koch et al., 1963, Wood et al., 1967; Hutagalung et al., 1968; Garrison et al., 1966). Because of this thyroid effect, Jahreis et al. (1986) co-administered iodine with nitrate supplementation. Other authors were only interested in the effects of nitrate on vitamin A levels and did not use a co-administration treatment to examine effects (Garner et al., 1958; Leĭtis and Emel′ianov, 1969). Because vitamin A inclusion is considered part of a healthy sow diet, these co-administrations were not considered confounding.

Leĭtis and Emel′ianov (1969) reported a 56% decrease in serum vitamin A at 366 mg/kg-bw/day of nitrate following a 4-wk treatment and, in this same study, reported a NOAEL for MetHb of 442 mg/kg-bw/day (no evidence of clinical effects or manifestation). Interestingly, the value chosen by EFSA of 410 mg/kg-bw/day from the 84-d study by Wood et al. (1967) also reported a decreased liver vitamin A value (73%) but still considered the study to represent an appropriate benchmark for swine nitrate safety. Koch et al. (1963) exposed animals to a very high level of nitrate (1.3 g/kg-bw/day) for 70 d, but because this study was assigned a K4 rating, it is not given much weight in terms of confidence. The WOE from this endpoint category suggests that the benchmark value established by EFSA remains appropriate, as the EFSA study (Wood et al., 1967) is part of the work that examined this potentially adverse endpoint. Additionally, the studies that supplemented with vitamin A or β-carotene suggest that much higher nitrate values (~870 mg/kg-bw/d) can be supported (Hutagalung et al., 1968).

#### Decreased growth performance.

Performance parameters typically consider decreased weight gain and decreased food intake to be signs of adversity that would be concerning to swine owners. Three studies reported different findings at relatively high exposure values (>1 g/kg-bw/day). Jahreis et al. (1986), a K2 study, determined a LOAEL of 1.4 g/kg-bw/day (for decreased weight gain and feed intake). This was a co-exposure study in which iodine was co-administered with potassium nitrate. Koch et al. (1963), a co-administration study with sodium nitrate and vitamin A, indicated a LOAEL of 1.3 g/kg-bw/day, but this study was scored as a K4 for quality and consequently given much less weight. Conversely, another K4-scored study (Tollett et al., 1960) found no change in weight gain in swine administered nitrate at levels equal to or less than 1.1 g/kg-bw/day. The K2-rated study by Hutagalung et al. (1968) found no effects of nitrate administration on weight gain at 870 mg/kg-bw/day following an 80-day treatment duration. β- Carotene was also co-administered in this study, but findings from this research lend confidence to the EFSA benchmark of 410 mg/kg-bw/day as being protective of effects on weight gain and food intake, and because methemoglobin levels were measured by Hutagalung et al. (1968) following a longer exposure duration, the study suggests that exposures upward of 870 mg/kg-bw/day would not be associated with MetHb concerns.

#### Decreased reproductive performance.

The effect of nitrate on reproductive health was the most sensitive (i.e., lowest dose) endpoint reported by Garner et al. (1958). This study was designated as K4 considering that the only information available came from an abstract from 1958, and, thus, the reliability is extremely low in terms of whether it warrants concern. One other study (Case, 1957) suggested a much higher LOAEL (883 mg/kg-bw/day), but this study was also scored as a K4 for the same reasons as Garner et al. (1958). Conversely, recent work by van den Bosch et al. (2019a, 2019b) and both Cargill, Inc. (2017, 2020) unpublished reports suggest that low-level supplementation has reproductive performance benefits, with no evidence of adverse events, at ranges of 19 to 334 mg/kg-bw/day, respectively. Such work and lack of emergence of recent, well-documented studies further corroborate that concern is not warranted with regard to adverse reproductive effects, at least at the levels reported in Fig. 1.

### WOE Evaluation Conclusions

The weight of the available evidence supports a lack of adverse health effects in endpoints including methemoglobin, vitamin A levels, growth and reproductive performance, and overall swine health, at estimated nitrate intakes from feed or drinking water that are much higher than the benefit of nitrate exposure values of 19 mg/kg-bw/day in swine (van den Bosch et al., 2019a, 2019b). The majority of the K1/K2-scored studies support, or are consistent with, EFSA’s findings that a NOAEL for nitrate supplementation is on the order of at least 400 mg/kg-bw/day. K1/K2-scored studies also consistently found no evidence for increased methemoglobin in the blood (when this endpoint was measured) or being associated with the manifestation of MetHb at nitrate exposure levels from 400 mg/kg-bw/day and up to 870 mg/kg-bw/day for 80 d (Garrison et al., 1966; Wood et al., 1967; Hutagalung et al., 1968; Leĭtis and Emel′ianov, 1969; Anderson and Stothers, 1978; Bouwkamp and Counotte, 1988; Sørensen et al., 1994; Cargill, Inc. 2017, 2020, unpublished data). One additional aspect of this work was to understand the variance around the EFSA benchmark value for nitrate safety in swine. The WOE evaluation presented herein provides confidence by using high-quality, reliable studies to support a conclusion that the NOAEL for nitrate is likely to be higher than the EFSA benchmark of 410 mg/kg-bw/day. Using studies with K1/K2 scores and methemoglobin blood measurements, we believe there is moderate-to-high confidence that the NOAEL for nitrate supplementation is most likely between 674 and 870 mg/kg-bw/day (~600 to 800 mg/kg-bw/day; Hutagalung et al., 1968; Trevisi et al., 2011).

## DISCUSSION

Older reports in the literature appear to be the source of historical concerns regarding sudden death or reproductive harm due to exposure to nitrate from nitrogen fertilizers, contaminated corn silage, well water, grasses, and grains (Gwatkin and Plummer, 1946; Case, 1957; Garner et al., 1958; Bjornson et al., 1961). These reports make alarming statements indicating that death can occur in pigs without any outward signs, attributable to nitrate contamination and its untoward effects. In contrast, recent work suggests that nitrate supplementation offers benefits to piglet livability (van den Bosch et al., 2019a, 2019b) in low-dose supplemental exposure scenarios. Our own efforts to identify the safe level of exposure to nitrate revealed a clear need for more transparency and documentation in the public domain on this topic. When sorting through the literature related to nitrate exposures associated with risk vs. benefits in swine, whether reviewing as a casual reader or a reader with a distinct need to clarify safety benchmarks, the literature proved difficult to review with confidence and clarity. A large number of publicly available studies investigating nitrate toxicity are more than 50 yr old and were published pre-GLP or in abstract or abbreviated formats only.

The work by EFSA (2009, 2020) helped to identify a potential benchmark value for nitrate safety in swine, but EFSA noted the weakness in having looked at only a few studies, leaving the educated reader with the same concerns when considering old case reports and suggestions of toxicity from reviews. It is possible that EFSA did not address the challenge of reviewing all available literature, given that normalizing the data across all swine nitrate ingestion studies proved to be a labor-intensive task. Further, such a comparison could not be conducted easily by a reader reviewing only a few of these studies to understand potential nitrate concerns. With these challenges in mind, the current review adds value to the publicly available literature, by providing the ability to identify oral (dietary or drinking water) nitrate exposure levels associated with health effects that have been reported in the literature, with a focus on understanding the risk of MetHb in swine.

The strength of the approach utilized in this review, as compared with previous reviews, was the use of modified Klimisch scoring for quality, data visualization for transparency, and a normalization procedure for nitrate dosing, so that studies could be objectively compared with each other. One of the major findings from this review determined that older studies that we believed were the impetus for perceived nitrate concerns (even at low levels) were of poor quality and lacked detail and, thus, should not weigh heavily in a WOE evaluation. This conclusion was supported by the fact that none of the results produced by recent studies corroborate the older reported findings. Additionally, none of the studies considered in this evaluation confirm evidence of MetHb resulting from the intentional administration of nitrate. Using the NOAELs and LOAELs from the higher-quality studies, it can be suggested that nitrate doses up to 870 mg/kg-bw/day produced no increases in methemoglobin measurements, further validating EFSA’s NOAEL of 410 mg/kg-bw/day nitrate as a safe benchmark.

One data gap based on the existing literature is the lack of clarification with regard to background nitrate. It would be helpful to understand with confidence the actual contributions of nitrate from diet and water. Generally speaking, nitrate is known to be present in drinking water. The Canadian Task Force on Water Quality (CTFWQ, 1987) recommends limits for nitrite-nitrogen (nitrite-N) and nitrate-nitrogen (nitrate-N) of 10 and 100 ppm, respectively, in water for livestock and poultry (Nyachoti and Kiarie, 2010). This is ~62 mg nitrate/kg-bw/day, assuming 0.14 L/kg-day intake in gestating and lactating swine. Adverse effects determined from our literature review were observed in swine after exposure to nitrate in drinking water at concentrations up to 2,000 ppm (~300 mg/kg-bw/day) for at least 6 wk (Seerley et al., 1965; Sørensen et al., 1994).

In addition, it is worth noting that the lack of MetHb observed may also relate to swine being less likely to metabolize nitrate to nitrite, which is required for the manifestation of MetHb. There seems to be a lack of clarity around risk in livestock related to interspecies differences in the rate of MetHb formation. EFSA did report that their review found differences mainly related to the extent and rate of nitrate reduction to nitrite, which is highest in ruminants due to the conditions in the rumen (relatively high pH and densely populated microflora; EFSA, 2020). Susceptibility to MetHb is lower in nonruminant, monogastric species, such as humans and swine, in part because the conversion of nitrate to nitrite occurs mainly in the large intestine, near the end of the digestive tract, where there is less chance for nitrite to be absorbed into the blood (Yaremcio, 1991; Sidhu et al., 2014; Thompson, 2014; EFSA, 2020). This type of information lends confidence to our finding that MetHb was of no risk at the nitrate administration ranges reported in the literature from high-quality studies.

In addition to swine, a plethora of peer-reviewed studies have examined the health effects of ingested nitrate in monogastric laboratory animal species across a wide array of hazard endpoints, such as toxicokinetics, acute/subacute toxicity, subchronic toxicity, reproductive and developmental toxicity, mutagenicity and genotoxicity, and chronic toxicity/carcinogenicity. These reports include studies conducted in rats, mice, dogs, and rabbits (e.g., Maekawa et al., 1982; Walker, 1990; Bryk et al., 2003). In addition, a substantial body of literature documents nitrate health impacts in humans, including case (generally involving acute exposures), clinical, and epidemiology studies. These studies in nontarget species, while outside the scope of this review, have been reviewed by authoritative bodies and regulatory agencies as the basis for establishing health-based guidance values. These studies also provide a firm foundation upon which the safety of nitrate and nitrite can be based in relation to the proposed use of calcium nitrate in food-producing animals (USEPA, 1991; JECFA, 2002; EFSA, 2008; IARC, 2010; WHO, 2011). Furthermore, in recognition that consumers of livestock products, such as pork, may be exposed to nitrate, the residual amount of nitrate in swine tissue is negligible, whether resulting from nitrate exposure through typical feeds and forages consumed by livestock or after supplemental dietary exposure. In their 2009 and 2020 assessments of nitrites in animal feed, EFSA considered the accumulation of nitrate and/or nitrite in animal products to be low, due to rapid metabolism and excretion.

The potential hazards of nitrate exposure are not unique to swine, as they have been investigated and observed in other livestock species, whether ruminant or monogastric. Considering the indications that low-dose nitrate in the diet may improve the livability of piglets in the often difficult farrowing process (van den Bosch et al., 2019a, 2019b), it is important to attain a more complete understanding of the risk, to determine whether the benefits can be achieved under acceptable risk scenarios. Our work suggests a high level of confidence in the value suggested previously by EFSA, the NOAEL of 410 mg/kg-bw/day. The WOE evaluation reviewed herein corroborates EFSA’s safety level for nitrate in swine as protective of concerns for MetHb and for adversity related to vitamin A depletion, growth, and reproductive performance. Moreover, the application of a robust methodology to consistently and objectively associate dietary nitrate with health and performance outcomes in swine provides moderate-to-high confidence that the NOAEL more likely resides above EFSA’s value and is probably between 674 and 870 mg/kg-bw/day (~600 to 800 mg/kg-bw/day) as determined by the 2-wk and 80-d nitrate studies by Trevisi et al. (2011) and Hutagalung et al. (1968), respectively. Additional research investigating blood methemoglobin levels and oxygen-carrying capacity will add to the body of evidence to further support and likely increase nitrate’s current NOAEL in swine.

## Supplementary Material

txab203_suppl_Supplementary_Table_S1

txab203_suppl_Supplementary_Data_S1
