# Supplementary material for: Review of potential risks associated with supplemental dietary exposure to nitrate-containing compounds in swine—a paradox in light of emerging benefits
Source: Transl Anim Sci. 2021 Oct 18;5(4):txab203. doi: 10.1093/tas/txab203 (PMC8665216; doi:10.1093/tas/txab203)
Supplement: txab203_suppl_Supplementary_Table_S1 [file txab203_suppl_supplementary_table_s1.docx]

**Supplementary Table S1. Summary of relevant nitrate studies in swine.**

| **Study Citation** | **Study Objective** | **Dose(s) and Form of Nitrate Administered** | **Swine Lifestage at Exposure/ Exposure Type/** | **Exposure Duration** | **Summary of Endpoints Examined and Results** | **POD and normalized POD^1^** | | | **Klimisch Score^2^** |
| --- | --- | --- | --- | --- | --- | --- | --- | --- | --- |
|  |  |  |  |  |  | **Observed Study NOAEL/ LOAEL and Basis** | **LOAEL Value Normalized to NO_3_ mg/kg-bw/day (ppm)** | **NOAEL Value Normalized to NO_3_ mg/kg-bw/day (ppm)** |  |
| **Dietary Studies Alphabetical by Author** | | | | | | | | | |
| Cargill, Inc., 2017, unpub-lished data | Identify potential negative effects of supplemental dietary nitrate on feed intake,  Performance, and blood methemoglobin (MetHb) levels in a dose-response study in piglets | 0, 0.2, 0.4, 0.6, 0.8, or 1.0% as CaNO_3_ | 22-day-old piglets (weaning Day 1 – Day 21) (n=12 per treatment group) | 21 days | No effects on weight gain, feed intake, and methemoglobin (highest level <2% at highest dose) | Not applicable | None | >334  (10,000) | 1 |
| Cargill, Inc., 2020, unpub-lished data | Investigate the effects of up to 6,000 ppm supplemental dietary nitrate on sow tolerance, observation of methemoglobinemia signs, or negative impacts on average daily feed intake | 0, 1200 or 6000 ppm as CaNO_3_ | Sow, GD 108 through lactation (n=8 per treatment group) | 27 days | No effects on sow and piglet weight gain and feed intake, hematology including methemoglobin in sows and piglets (highest level of 1.3% and 0.9%, respectively, at high dose), serum calcium, phosphate and bone measures, reproductive performance | Not applicable | None | >135  (6,000) | 1 |
| Case, 1957, published as abstract only | Review of case reports from NO_3_-contaminated feed | Case reports of 5.52% NO_3_ in rape and 0.53% NO_3_ in oats, unspecified salt form(s) | Sows  (replicate number per treatment group not specified) | Unspecified | Case reports of abortion, piglet mortality, offspring abnormalities, with decreased serum NO_3_ and vitamin A (data not reported) | Abortion, major offspring effects, and decreased serum vitamin A | 883  (55,200) | None | 4 |
| Garner  et al., 1958, published as abstract only | Assess effects of nitrate on reproduction and vitamin A storage | 0.0, 0.5, 1.0, or 2.0% as KNO_3_ | Sow, 35 days after breeding possibly through lactation (n=2 per treatment group) | Up to 101 days | Dose-related increase in serum NO_3_; reduced “livability” and number of strong piglets with higher levels of nitrate, vit A deficiency in one litter of sows at 2%; reduced liver vit A in piglets; no effect on litter size or sow milk production | Insufficient information to determine^3^ | ---^3^ | ---^3^ | 4 |
| Hutagalung et al., 1968 | Investigate the effect of nitrate and nitrite in feed on “carotene utilization” in treated pigs | 0, 0.75, 1.5, or 3% as KNO_3_ with β-carotene (3520 IU/kg feed) or vitamin A (1173 IU/kg feed) | Growing (26 kg) pigs (n=8 per treatment group) | 80 days | Reduced weight gain (~21%, p<0.01) at 3% NO_3_ (for both vitamin A or β-carotene groups), no effects on serum and liver vitamin A, methemoglobin (highest level of 0.68 g/100 mL at 3% NO_3_ with β-carotene), hemoglobin, and hematocrit | Reduced weight gain (21% with β-carotene 3520 IU/kg feed or 23% with vitamin A 1173 IU/kg feed) | 1,740  (30,000) | 870  (15,000) | 2 |
| Jahreis  et al., 1986 | Examine the influence of NO_3_ on thyroid hormone concentration and somatomedin-C (Sm-C) serum concentration in the presence of iodine supplementation | 0 or 3% as KNO_3_ with or without 0.8 mg/kg iodine supplementation following week 5 of exposure | Growing (42-day-old) pigs (n=9 per treatment group) | 6 weeks (with additional iodine supplemen-tation following week 5) | Decreased weight gain (38%), feed intake (30%), thyroxine (T_4_), and reverse triiodothyronine (rT_3_), without effects on T_3_ and Sm-C after 5 weeks of exposure; levels of T_4_ and rT_3_ were restored to levels without statistical significance as compared to control following iodine supplementation in week 6 of the study | Decreased weight gain (38%), feed intake (39%), T_4,_ and reverse T_3_ | 1,446  (18,300) | None | 2 |
| Jahreis  et al., 1987 | Examine the effect of acute and sub-acute dietary NO_3_ supplementation before and after iodine supplementation on the concentration of T_4_, T_3_, and Sm-C | 0 or 3% as KNO_3_ with or without 0.5 mg/kg iodine supplement | Growing (56-day old) (n= 7 per treatment group) | 2 days or 6 weeks | Without (but not with) iodine supplementation, NO_3_ feeding decreased weight gain (39%), feed intake (22%), and T_4_ and Sm-C levels, and increased serum NO_3_ and methemoglobin (0.07 vs 0.03 in controls, units not reported, p<0.01) at 6 weeks (but not 2 days); no effects on T_3_ | Decreased weight gain (39%), feed intake (22%), T_4_, Somato-medin C, and increased methemoglobin | 1,354  (18,300) | None | 2 |
| Koch et al., 1963, published as abstract only | Assess the effects of different levels of sodium nitrate and vitamin A supplementation on growing swine | 0 (>400 IU vitamin A/pound feed), 0.6-1.0, or 1.2-2.0% as NaNO_3_ | Growing pigs (total sample size=14; replicate number per treatment group not specified) | 70 days | No effects on weight gain, feed efficiency, hemoglobin, or methemoglobin (highest level of 2.52%); non-dose-related serum vitamin A decreased same magnitude at both concentrations (~50% of controls) | LOAEL: 0.6-1.0%  Decreased serum vitamin A (50%) (on vitamin A supplemented diet, >400 IU/pound) | 386  (6,132) | None | 4 |
|  |  | 0, 3.0 or 5.0% as NaNO_3_ with 2000 IU vitamin A/pound feed or 3.0% NaNO_3_ with 1000 IU vitamin A/pound feed | Growing pigs (total sample size=20; replicate number per treatment group not specified) | 81 days | Decreased weight gain (50%) and liver but not serum vitamin A (35%); no effects on feed efficiency, hemoglobin, methemoglobin (highest level of 3.35% at 3% NaNO_3_) | Decreased weight gain (50%) and liver vitamin A (35%) on vitamin A supplemented diet (2000 IU/pound) | 1,270  (21,900) | None |  |
| Leĭtis and Emel'ianov, 1969 | Clarify the effects of nitrates on the health and productivity of pigs | Gradual weekly increase from 0.3 to 1000 mg NO_3_/kg bw/day as KNO_3_ | Growing pigs (4-mo old) (n=3 per treatment group) | 1 to 4 weeks | No clinically noticeable effect on general condition (e.g., body temperature, appetite, behavior, weight gain), increased methemoglobin (highest level of 11.4% at highest dose following week 4 of dosing), decreased serum vitamin A (~56%) following week 4 of dosing, and increased urine NO_3_ and NO_2_ | Decreased serum vitamin A (56%) (barley/feed with unknown vitamin A content) | 366  (vitamin A)  None  (metHg) | 183  (vitamin A)  442^4^  (metHb) | 2 |
| Paulson and Aschbach-er, 1990 | Evaluate the effect of feeding nitrate-fortified diets for 3 weeks on the disposition of Sulmetin swine | 0, 10, 100, 500 or 1000 ppm NaNO_3_ and 110 ppm Sulmet, sodium sulfamethazine, a sulfa antibiotic | Growing crossbred swine (58-74 kg) (n=6 per treatment group; sex unknown) | 21 days | No effects on daily weight gain; increased NO_2_ in oral cavity at $\geq$500 ppm NO_3_ | Not applicable (co-exposure with antibiotic) | None | >40  (1000) | 3 |
| Trevisi  et al., 2011 | Assess the dose-dependent effects of dietary nitrate on levels of recirculating nitrate in the saliva and the conversion of nitrate to nitrite, if nitrate recirculation into the saliva is altered in a time-dependent manner (i.e., compare dietary supplementation of 1 or 2 weeks), and if changes in oral microbiota are time-dependent | 0 or 1.22% as KNO_3_ | Growing (42-d old piglets) (n=6 per group) | 2 weeks | No effects on growth and feed intake; 8-fold increase in salivary NO_3_ through end of study, while serum NO_3_ and salivary NO_2_ peaked on Day 7 of exposure; significant difference also observed in Shannon’s index of microbiota diversity in saliva in control vs. KNO_3_ treated animals | Not applicable | None | >674  (7,442) | 1 |
| Tollett  et al., 1960, published as abstract only | Assess the effects of nitrate on growth and reproductive performance over four trials assessing effects of either KNO_3_ or NaNO_3_ with or without coadministration of vitamin A | NO_3_ dose and form (KNO_3_ or NaNO_3)_ unspecified | Gilts (n=24; n per group unknown; Trial 4 specifically assessed reproductive performance) | Unspecified | Reduced weight gain (p<0.01, unspecified magnitude); no effects on corpora lutea number, percent implantation, ovary, embryo, placenta, or thyroid weights | Reduced weight gain | --^5^ | --^5^ | 4 |
|  |  | 0, 0.61, 1.23, 1.84, 2.4, or 3.17% NO_3_ as KNO_3_ | Growing pigs  (n=7 per group; Trial 1) | Unspecified | Reduced weight gain (p<0.05, unspecified magnitude) | Reduced weight gain | 1,440  (24,000) | 1,104  (18,400) |  |
|  |  | 3.17% NO_3_ as KNO_3_ or NaNO_3_ | Growing pigs  (n=8 per group; Trial 2) | Unspecified | Reduced weight gain (p<0.01, unspecified magnitude) | Reduced weight gain | 1,902  (31,700) | None |  |
|  |  | 0, 0.61, 1.84, or 3.17% NO_3_ as KNO_3_ with vitamin A 1500 or 3600 IU/pound | Growing pigs  (n=6 per group; Trial 3) | Unspecified | Reduced weight gain (p<0.1, unspecified magnitude) and methemoglobin (unspecified) | Reduced weight gain and increased methemoglobin on vitamin A supplemented diet (1500 or 3600 IU/pound) | 1,902  (31,700) | 1,104  (18,400) |  |
| van den Bosch et al., 2019a | Evaluate potential beneficial effects of maternal dietary nitrate supplementation on reproductive performance of sows and piglet performance | 0, 0.03, 0.06, 0.09, 0.12 or 0.15% NO_3_ as CaNO_3_ | Gilts and Sows, GD 108 to LD 5 (n=47-52 per dose group) | 12 days | No adverse effects on sow body weight, reproductive performance, litter characteristics, piglet body weight, vitality, and survival (several parameters were affected by nitrate supplementation in a beneficial direction) | Not applicable | None | ≥15–19^6^ (1,500) | 1 |
| van den Bosch et al., 2019b | Investigate potential effects of maternal dietary nitrate supplementation and subsequent nitric oxide (NO) formation on duration of farrowing, piglet asphyxiation, vitality at birth, and/or loss of potential viable piglets | 0, 0.03, 0.06, 0.09, 0.12 or 0.15% NO_3_ as CaNO_3_ | Sow, GD 108 to LD 5 (n=27-32 per group) | 12 days | No adverse effects on sow feed intake, reproductive performance, placental health, piglet health, and blood gas acid-base parameters (pH, pO2, pCO2, BEecf, HCO3, sO2, lactate) and NO_3_ in umbilical-cord blood | Not applicable | None | >16^6^  (1,500) | 1 |
| **Drinking-Water Studies Alphabetical by Author** | | | | | | | | | |
| Anderson and Stothers, 1978 | Evaluate the effects of saline water (6000 ppm concentration based on dissolved sulfate) alone or in combination with nitrate nitrogen (N-NO_3_) | 0, 100 or 300 ppm as N-NO^3^ unspecified salt form with 6000 ppm sulfate | Young weanling pigs, (n=9-18 per group) | 3 or 6 weeks | No effects on feed and water intake, body weight/gain, scouring days, methemoglobin (highest level of 0.24%), liver vit A level, kidney size | Not applicable | None | >252  (1328) | 2 |
| Bjornson et al., 1961 | Review of case reports of KNO_3_ in swine farm drinking-water wells | KNO_3_ in 4 of 6 wells from 200-510 ppm KNO_3_ (122-311 ppm NO_3_) | Not specified – 4 pigs noted in one case study as affected. | Acute | Mortality in 4 out of an unspecified number of farm pigs; unknown which of four wells the affected pigs drank from | Mortality | 12  (122) | None | 3 |
| Bouwkamp and Counotte, 1988 | Assess the effects of increased concentrations of nitrate in drinking water of weaned piglets” | 0 or 200 mg/L NO_3_ as KNO_3_  (Trial 1) | Weaned piglets (9 kg bw until 21 kg bw) (n=116 or 105 per group) | From 9 kg bw until 21 kg bw | No effects on piglet growth | Not applicable | None | >40  (200) | 2 |
|  | Preliminary study regarding the effects of increased concentra-tions of nitrate in the drinking water of fattening pigs | 0, 100, 200, or 500 mg/L NO_3_ as KNO_3_  (Pilot Trial) | Growing pigs (age/weight not specified) (n=4 per NO_3_ group, n=20 controls) | 79 days during “entire fattening period” | No effects on pig grow  th or hemoglobin, methemoglobin (highest level of 5.2% at 200 mg/L on day 79) | Not applicable | None | >76  (500) | 2 |
|  | Additional assessment of the effects of increased concentrations of nitrate in the drinking water of fattening pigs | 0 or 500 mg/L NO_3_ as KNO_3_  (Trial 2) | Growing pigs (age/weight not specified) (n=70 per group) | “Entire fattening period” | No effects on pig growth, hemoglobin, and methemoglobin (highest level of 1.6%) | Not applicable | None | >76  (500) | 2 |
| Bruning-Fann et al., 1996 | Determine associations between nitrate observed in farm well water used as swine drinking water, and farrowing swine health and productivity as part of the National Swine Study | 0 to 460.6 ppm NO_3_ in well water among 571 farms | Gestating and lactating sows (n=27,201) | 3-month prospective epidemiol-ogy | No effects on sow and piglet health, reproductive performance, litter characteristics | 75%ile NO_3_ concentration (18.5 ppm) of 571 farms; no effects observed | None | >2.4  (18.5) | 1 |
| Persson et al., 1988 | Investigate the potential mutagenic effects of high levels of nitrate in drinking water | <1 mg/L (control) or 100 mg/L  NO_3_, unspecified salt form | Growing pigs (n=19-23 per group) | Unspecified | No increases in micronuclei in lymphocytes and bone marrow and lack of mutagenicity of urine incubated with *S. typhimurium* TA98 and *E. coli* WP2 uvRA | Not applicable | None | >10  (100) | 2 |
| Seerley et al., 1965 | Assess the effects of drinking water supplemented with NaNO_3_ on growing-finishing pigs | 0, 50, 125, or 300 ppm NO_3_-N as NaNO_3_ | Growing pigs (47 kg) (Trial 1, n=6 per group) | 61 or 97 days | No effects on weight gain, food or water intake | Not applicable | None | >146  (1,328) | 2 |
|  | Assess the effects of drinking water supplemented with NaNO_3_ on gilts from weaning through gestation and lactation | 0 or 300 ppm NO_3_-N as NaNO_3_ | Gilts (20.9 kg) and gestating and lactating sows (Trial 3, n=8 per group) | Up to 125 days | No effects on body weight, feed intake, serum vitamin A, or “thriftiness” of gilts; no effects on reproduction through two farrowing periods | Not applicable | None | >186  (1,328) |  |
| Sørensen et al., 1994 | Examine whether intake of drinking water  containing supplemental nitrate and/or manure-derived nitrite had detrimental effects on performance and blood methemoglobin in young pigs after early weaning | 0, 50, 250, 1000 or 2000 ppm NO_3_ as KNO_3_ (excluding groups also exposed to manure in water) | Early weaned (3 wk old 6 kg) or growing pigs (n=10 per group) | 6 weeks or until slaughter | No effects on methemoglobin, weaning diarrhea, growth rate, feed intake/utilization, water consumption or blood hemoglobin | Not applicable | None | >300  (2,000) | 2 |
| Wood et al., 1967; Garrison et al., 1966 (pre-publica-tion of Wood et al., 1967 work) | Address the effects of adding NO_3_ as KNO_3_ to drinking water on the utilization of carotene by growing-finishing swine | 0, 0.075, 0.150, or 0.300% as KNO_3_ with β-carotene (3520 IU/kg feed) supplement or 0.300% KNO_3_ with vitamin A palmitate (1173 IU/kg feed) supplement | Growing (24.7 kg) (n=8 per group) | 84 days | Decreased liver vitamin A on β-carotene 73%, p<0.01) but not vitamin A palmitate (55%, p>0.01)-supplemented diet; no effects on weight gain, feed intake, blood vitamin A, hemoglobin, methemoglobin, and hematocrit | Decreased liver vitamin A (73%) on β-carotene supplemented diet (3520 IU/kg) | 450^7^  (3,000) | 225  (1,500) | 2 |
| **Gavage Studies** | | | | | | | | | |
| Gwatkin and Plummer, 1946 | Assess potential for KNO_3_-induced salt poisoning in swine | KNO_3_ from 569 to  6825 mg NO_3_/kg bw/day | Piglet (6-15kg)  (six dose levels, n=1 per dose) | one dose | Mortality, general examination, including gross and histological pathology | Death, gastrointestinal lesions (n=1) | 1,128 (not applicable) | 644 (not applicable) | 3 |

Table abbreviations: NOAEL = no-observed-adverse-effect-level; LOAEL = lowest-observed-adverse-effect-level; POD = point-of-departure; bw = body weight

^1^ NOAEL and LOAEL dose conversions to mg nitrate/kg-bw/day based on (1) study-specific body weight and feed intake (diet studies) or water intake (drinking-water studies) data, if reported, (2) a surrogate study with a comparable design, as indicated, or (3) default assumptions in Table 2, if study-specific data were not reported (See Supplementary Information)

^2^ Modified score of 1=Reliable, 2=Reliable with restriction, 3=Unreliable, or 4=Unassignable (see Methods).

^3^ Data, statistical significance or affected dose level(s) not reported; lowest concentration of 0.5% = 41 or 48 mg/kg-bw/day (estimated intake depends on assumptions for parity = 1 or 2) (Garner et al., 1958).

^4^ Adjusted dose over 4-week exposure, based on 300 mg/kg bw/day (Week 1), 600 mg/kg bw/day (Week 2), and 1000 mg/kg bw/day (Weeks 3 and 4). Increased metHg (11.4%) at Week 4 not considered adverse, because not associated with clinically noticeable effects on general condition (Leĭtis and Emel'ianov, 1969).

^5^ Dose level(s) for Trial 4 not reported (Tollet et al., 1960).

^6^ EFSA (2020) estimated NOAEL to be 19 mg/kg-bw/day (based on unspecified assumptions) compared to 15 or 16 mg/kg-bw/day estimated in the present assessment (van den Bosch et al., 2019a,b; see Supplementary Information).

^7^ Estimated to be 410 mg nitrate/kg-bw/day, assuming 11.5 L/day water intake, and considered NOAEL by EFSA (2020) (Wood et al., 1967).
